# MOLLI T_1 _mapping versus T_2 _W-SPAIR at 3T: myocardial area at risk measurements and the influence of microvascular obstruction

**DOI:** 10.1186/1532-429X-16-S1-O22

**Published:** 2014-01-16

**Authors:** Donnie Cameron, Nishat Siddiqi, Christopher J Neil, Baljit Jagpal, Margaret Bruce, Andrew Richardson, Thomas W Redpath, Michael P Frenneaux, Dana K Dawson

**Affiliations:** 1 University of Aberdeen, Aberdeen, UK; 2 The Queen Elizabeth Hospital, Adelaide, South Australia, Australia

## Background

Robust CMR imaging is required for the delineation of myocardial area at risk (AAR), so that the success of reperfusion therapies can be evaluated. In this work, we investigate the performance of T_1 _mapping in assessing AAR one week post-STEMI, and explore the effect of microvascular obstruction (MVO) on T_1 _relaxation times.

## Methods

CMR imaging was conducted on a Philips 3T Achieva MRI scanner. T_2_W-weighted spectral attenuated inversion recovery (T_2_WW-SPAIR), modified look-locker inversion recovery (MOLLI) T_1 _mapping and late gadolinium enhancement (LGE) sequences were applied as short axis stacks in 10 healthy volunteers and 62 STEMI patients. Receiver operator characteristic (ROC) analysis was applied to calculate a cut-off T_1 _to to discriminate AAR from normal myocardium. The presence of LGE was used as the positive ROC test state, while healthy myocardium, as measured in volunteers, was used as the negative ROC test state. For comparison with T1 mapping, the AAR was also measured on T_2_WW images using a threshold signal intensity > 2SD greater than remote. The derived myocardial edema volumes and salvage indices were compared between MVO+ and MVO- groups.

## Results

**Figure 1:**
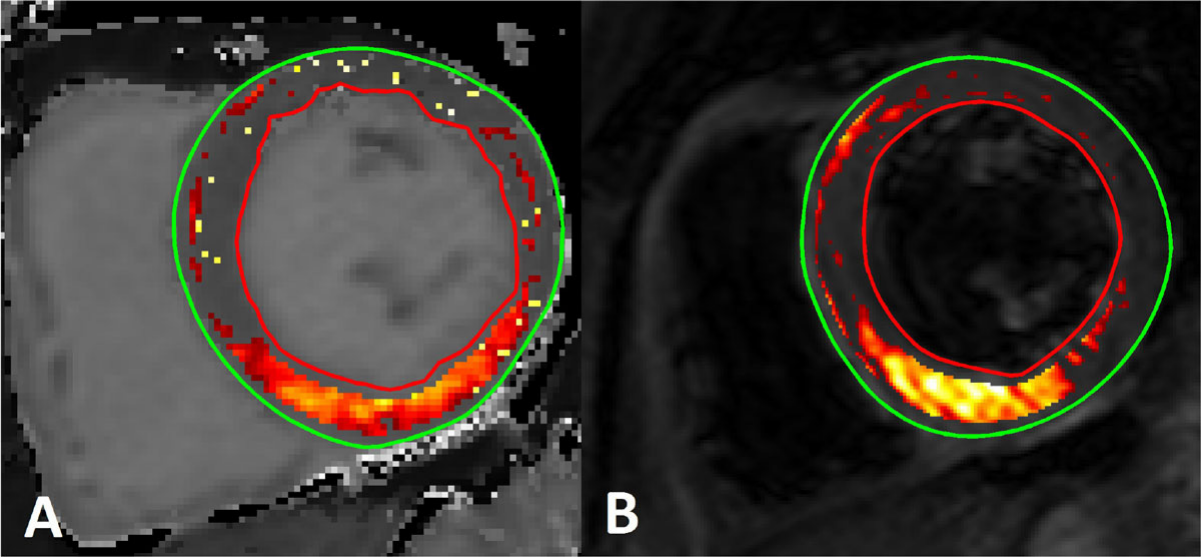
**The area at risk as delineated by (A) MOLLI T_1 _mapping and (B) T_2_-weighted SPAIR**.

For T_1 _mapping, ROC analysis gave a significantly larger area-under-the-curve (AUC) as compared to T_2_WW-SPAIR for delineating myocardial edema (AUC = 0.89 vs 0.83, p = 0.009) as well as better sensitivity/specificity (83/83% vs 73/73%). Neither method was significantly affected by the presence of MVO. The calculated ROC cut-off for T_1 _mapping was 1243 ms, and this gave a significantly larger AAR than that measured with a T_2_W-SPAIR 2SD threshold (p = 0.006). Using the T_1 _mapping cut-off, patients with MVO had a significantly larger AAR and a poorer salvage index than patients without MVO (p < 0.05 for both). The AAR measured using each of the two methods is illustrated in Figure 1, and AAR and salvage index measurements are shown in Table 1.

**Table 1:** Area at Risk and Salvage Index

Method	All Patients	MVO+	MVO-
T_2_W-SPAIR 2SD AAR Volume (%)	40 (16)	28 (11)†	48 (13)†
T_1 _Mapping ROC AAR Volume (%)	55 (7)*	57 (7)*	53 (8)
Salvage Index by T_2_W-SPAIR 2SD	0.66 (0.23)	0.75 (0.17)	0.59 (0.25)
Salvage Index by T_1 _Mapping ROC	0.73 (0.22)	0.89 (0.08)*†	0.63 (0.22)†


Area at risk volume and salvage index measured using T_2_W-SPAIR and T_1 _mapping - * denotes a statistically significant difference between T_1 _mapping and T_2_W-SPAIR techniques; † denotes a statistically significant difference between MVO+ and MVO- groups (p values in the text). Data is presented as mean (SD).

## Conclusions

T_1 _mapping at 3T can be used to automatically delineate AAR one week post-STEMI. It delimits larger volumes of edema and demonstrates less variability than T_2_WW-SPAIR. MVO did not significantly affect the discriminatory power of either of these techniques at seven days post-STEMI.

## Funding

This study was supported by a Medical Research Council (UK) grant, as a sub-study of Nitrites in Acute Myocardial Infarction, NCT01388504.

